# Empathy levels among undergraduate medical students in Karachi, Pakistan: a cross-sectional study

**DOI:** 10.1097/MS9.0000000000001042

**Published:** 2023-07-06

**Authors:** Masooma Naseem, Burhanuddin Tahir, Afia Salman, Sara Qadir, Rida Farhan, Sajjad Ali, Zehra Naseem, Warda Ahmed, Mahfuza Anan

**Affiliations:** a Ziauddin Medical College, Ziauddin University; b Dow Medical College, Dow University of Health Sciences; c Bangladesh Medical College, Dhaka, Bangladesh

**Keywords:** empathy, medical students, observational study, Pakistan, surveys and questionnaires

## Abstract

Empathy is a concept native to the field of medicine. However, over the course of medical school, empathy levels are expected to decline. The aim of this study was to assess the empathy levels among medical students in relation to their year of study and specialties of choice in the future.

**Materials and methods:**

A cross-sectional study was conducted to assess empathy levels among students from medical colleges in Karachi, Pakistan, using an online survey. The total duration of the study was 2 months. The authors analyzed the data using SPSS version 20.

**Results:**

A total of 463 undergraduate medical students participated in this survey. The overall mean empathy score was 101.9±16.3 with 104.6±14.1 for females, which was significantly higher than the male participants. The highest empathy levels were demonstrated in fourth-year medical students with a mean empathy score of 104.1±16.3, whereas, the lowest empathy levels were found in second-year medical students with a mean empathy score of 99.8±9.4. Study participants considering emergency medicine, neurology, obstetrics/gynecology, and oncology as their specialty of choice demonstrated the highest empathy levels followed by pediatrics and internal medicine.

**Conclusion:**

On average, there was a female preponderance in empathy levels among undergraduate medical students. Empathy levels among fourth-year students involved in clinical practice were greater as compared to students in their initial years of study. Further investigations are required to validate the findings of this study.

## Introduction

Highlights

- Empathy levels are expected to decline through the course of medical school.
- This cross-sectional study assessed empathy levels among undergraduate medical students in Karachi, Pakistan, in relation to the specialties of choice and the year of study.
- The study concluded relatively higher levels of empathy in female medical students.
- Higher empathy levels were observed in fourth-year medical students compared to students in the initial years of undergraduate study.
- Regarding the specialties of choice, greater empathy levels are observed in participants who chose emergency medicine, neurology, obstetrics/gynecology, and oncology.

### Background

The ambiguity associated with the definition of empathy and the absence of psychometrically efficient tools to measure empathy in a particular setting has led to a lack of empirical research on empathy in medical schooling and patient care. Nevertheless, empathy has received recognition from media, politics, business, arts, ethics, and academia, particularly in the training of healthcare professionals and patient interaction^1^.

Research studies recognize distress as a major factor affecting self-assessed empathy in medical students, trainees, and residents^2^. At the beginning of medical school, students and trainees have high values of idealism, enthusiasm, and humanity, but a decline is seen as they are confronted by the ground clinical reality of the ill health of the patients, the severity of diseases, and human suffering and death. This leads to a shift in their focal point and they are directed towards technological and objective aspects of medicine instead of humanistic approaches^3^.

Only one study from Karachi, Pakistan, has assessed empathy among first-year and final-year undergraduate medical students. The study found no significant differences in the empathy levels of first-year and final-year medical students, but the female to male ratio was relatively higher in terms of empathy levels^4^. A similar study conducted in India identified the factors associated with empathy in medical students. The study found a significant association of empathy with sex, with higher empathy scores in female participants. The study found no significant association of empathy with age, place of residence, choice of specialty, and decision for enrollment in the undergraduate program^5^. The present study aimed to evaluate the levels of clinical empathy and associated factors in undergraduate medical students at government and private universities in Karachi, Pakistan.

### Objectives

The objectives of this study include the assessment of clinical empathy levels among undergraduate medical students and the association of study year and specialty of choice with the empathy levels.

## Materials and methods

### Ethics statement

Prior to the conduction of this study, ethical approval was obtained from the Institutional Review Board. Consent was taken from the study participants prior to the acquisition of data. (Protocol reference number 0110721MNY5). The study has been registered in ClinicalTrials.gov (Identifier NCT05736276). The study has been reported in line with the strengthening the reporting of cohort studies in surgery (STROCSS) criteria^6^.

### Study design, setting, and participants

This descriptive cross-sectional study was conducted over a period of 2 months (1 June 2022–30 July 2022) and surveyed 463 participants from medical colleges in Karachi, Pakistan, belonging to both the government and private sectors. Undergraduate medical students enrolled in different years of the academic program were involved to cater to those in the preclinical years as well as those receiving clinical exposure.

Following approval from the institutional review committee, the sample size was calculated at a 5% confidence level. The CI was calculated at 95%, expecting the group difference of mean empathy score and SD by age and sex. Data regarding demographics and empathy scores were gathered via a self-administered questionnaire. The sample was recruited using a nonprobability convenience sampling technique.

The study used the Jefferson Scale of Empathy to evaluate clinical empathy levels among undergraduate medical students. This tool, developed in 2001, is a validated self-administered questionnaire comprising 20 questions to evaluate three factors: standing in the patient’s shoes, compassionate care, and perspective thinking. The total score range of the Jefferson Scale of Empathy is 20–140. Each factor of the questionnaire was attributed to both positive and negative phrased questions with ‘1’ signifying ‘strongly disagree’ and ‘7’ signifying ‘strongly agree’^7^.

### Statistical methods

The data was analyzed using SPSS 20. Empathy score with an advancing year of study was investigated using ANOVA. An ANOVA with post-hoc Tukey’s test was used to study the relationship between the year of study and empathy score. In addition, a *t*-test was used to assess the relationship of mean empathy score with sex and age group. For the purpose of analysis, scores were divided into below-average and above-average categories. A comparison of these categories with demographics was done using Pearson’s *χ* ^2^-test. A *P*-value of ≤ 0.05 was considered significant.

## Results

### Participants

Of the 463 participants, 82.3% (*n*=381) were females and 17.7% (*n*=82) were males. Of the total medical students, 58.1% (*n*=269) belonged to the age group of 22–24 while the remaining 41.9% (*n*=194) were less than 22 years old. Furthermore, when asked what specialty the students aimed to pursue in the future, 24.4% (*n*=113) responded with general surgery or subspecialties; 22.9% (*n*=106) showed interest in internal medicine or subspecialties, 3.5% (*n*=16) aimed toward emergency medicine, 12.1% (*n*=56) towards pediatrics; and 8.6% (*n*=40) chose neurosurgery. Concerning the remaining study participants, 12.1% (*n*=56) did not have a preference for a certain specialty, whereas 16.4% (*n*=76) opted for other options including family medicine, oncology, obstetrics/gynecology, neurology, orthopedic, and plastic surgery as demonstrated in Table 1.

**Table 1:** Study demographics

Characteristics	*n* (%)
Age (years)	
<22	194 (41.9)
22–24	269 (58.1)
Sex	
Male	82 (17.7)
Female	381 (82.3)
Year of medical school	
2nd year	64 (13.8)
3rd year	106 (22.9)
4th year	197 (42.5)
> 4th year	96 (20.7)
Career preference	
General surgery or subspecialties	113 (24.4)
Internal medicine or subspecialties	106 (22.9)
Emergency medicine	16 (3.5)
Family medicine/General practice	8 (1.7)
Neurology	16 (3.5)
Pediatrics	56 (12.1)
Neurosurgery	40 (8.6)
Obstetrics/Gynecology	8 (1.7)
Oncology	8 (1.7)
Orthopedic surgery	8 (1.7)
Plastic surgery	28 (6.0)
Undecided	56 (12.1)

Among subspecialties, 21.2% (*n*=98) showed keen interest toward cardiology, 8.6% (*n*=40) in nephrology, and 5.2% (*n*=24) planning to pursue hematology/oncology. The overall mean empathy score was found to be 101.9±16.3.

### Study results

The mean empathy level in the age group below 22 years was 103.5±13.0, while between the ages of 22–24 it was found to be 100.8±18.3. This difference was not significant (*P*=0.078) on applying a *t*-test. Females scored higher than males with a mean empathy score of 104.6±14.1 whereas, the mean empathy score in males was 89.2±19.7. This difference was significant with a *P*-value of 0.000 on applying a *t*-test. The highest empathy level was observed amongst fourth-year medical students, with a mean empathy score of 104.1±16.3. This was followed by third-year medical students with a mean empathy score of 101.2±15.4. The lowest empathy scores were found for the second-year (99.8±9.4) and for years of training higher than the fourth-year (99.8±20.2). When ANOVA was applied for the comparison of empathy scores between groups, no significant difference was found between any two particular years of training/study with a *P*-value of 0.09. The association of age group, sex, and year of study/training with the mean empathy score is demonstrated in Table 2.

**Table 2:** Association with mean empathy score

	Empathy score (Mean±SD)	*P*
Age (years)		0.078
<22	103.5±13.0	
22–24	100.8±18.3	
Sex		0.000
Male	89.2±19.7	
Female	104.6±14.1	
Year of training		0.09
2nd year	99.8±9.4	
3rd year	101.2±15.4	
4th year	104.1±16.3	
>4th year	99.8±20.2	

Scores were categorized into two groups: below-average (score<102) and above-average (score≥102). Pearson’s *χ* ^2^-test was applied to find associations between these groups with age, sex, year of study, and career preference. Among student groups of age less than 22 years, most (66%) had above-average empathy scores while within the 22–24 age group, approximately half (55.4%) had above-average empathy scores. Out of them, 66.4% of females had higher scores compared to 29.3% of males. The majority of fourth-year students (75.6%) had above-average scores followed by half of the third-year (52.8%) and greater than fourth-year participants (50%) as compared to only 37.5% of second-year students. Among the selected specialties as career preferences, below-average scores were seen in 100% (*n*=8) of the students who selected family medicine/general practice and orthopedic surgery followed by 80% (*n*=32) students who selected neurosurgery as their future specialty. All students who aimed to pursue emergency medicine, neurology, obstetrics/gynecology, and oncology demonstrated average or above-average empathy scores. Those who selected pediatrics, internal medicine, and surgery had relatively higher scores at 71.4% (*n*=40), 67.9% (*n*=72), and 63.7% (*n*=72), respectively. All associations were found to be significant as seen in Table 3.

**Table 3:** Categorization of empathy scores and association with demographics

	Empathy scores *n* (%)		
Characteristics	Below-average	Above-average	*P*
Age (years)			0.022
<22	66 (34)	128 (66)	
22–24	120 (44.6)	149 (55.4)	
Sex			0.000
Male	58 (70.7)	24 (29.3)	
Female	128 (33.6)	253 (66.4)	
Year of medical school			0.000
2nd year	40 (62.5)	24 (37.5)	
3rd year	50 (47.2)	56 (52.8)	
4th year	48 (24.4)	149 (75.6)	
>4th year	48 (50)	48 (50)	
Career preference			0.000
General surgery or subspecialties	41 (36.3)	72 (63.7)	
Internal medicine or subspecialties	34 (32.1)	72 (67.9)	
Emergency medicine	0 (0)	16 (100)	
Family medicine/General practice	8 (100)	0 (0)	
Neurology	0 (0)	16 (100)	
Pediatrics	16 (28.6)	40 (71.4)	
Neurosurgery	32 (80)	8 (20)	
Obstetrics/Gynecology	0 (0)	8 (100)	
Oncology	0 (0)	8 (100)	
Orthopedic surgery	8 (100)	0 (0)	
Plastic surgery	15 (53.6)	13 (46.4)	

## Discussion

This study aimed to assess the clinical empathy levels among undergraduate medical students and the association of study year and specialty of choice with the empathy levels.

A total of 463 undergraduate medical students participated in this survey. The mean empathy score was higher among the female participants as compared to their male counterparts. The highest empathy levels were demonstrated in fourth-year medical students, in contrast to low empathy levels among students in other years of study. This cross-sectional survey also highlighted the association between career preference and empathy levels. Study participants considering emergency medicine, neurology, obstetrics/gynecology, and oncology as their specialty of choice have the highest empathy levels, followed by internal medicine and pediatrics.

Our study focused on evaluating empathy with respect to variables of sex, year of study, and choice of future specialty training. It was identified that the overall mean empathy score was 101.9±16.3 which is significantly low compared to studies outside Pakistan that have suggested scores in the range of 104–110^8^. These significant differences may have aroused due to differences in sampling methodologies and the way different cultures define empathy. Medical institutions across various countries opt for different criteria for admission to medical college and follow different curriculums set to their cultural and traditional standards, which can all have an impact on the empathy score of students belonging to different countries and backgrounds^9^.

In our study, the second-year and final-year students scored the lowest on empathy followed by the third-year medical students. The highest score was observed in fourth-year students. These findings are consistent with previous studies, suggesting that there is a decline in empathy levels after exposure to clinics and toward the end of clinical training^10^. It has also been observed that increasing levels of burden and workload associated with an inappropriate sleep pattern and a lack of relaxation have also contributed to a decline in empathy levels in medical students. Mental exhaustion, stress, and extreme anxiety have proven to impact students’ empathy levels^2^.

In this cross-sectional study, female medical students demonstrated greater levels of empathy, with a mean empathy score of 104.6±14.1, than male medical students. This has been reported by another cross-sectional study by Shashikumar *et al.*, evaluating empathy among medical undergraduates. The potential reason underlying relatively higher empathy levels among females is that they are less influenced by factors responsible for diminishing empathy^11^.

Coherent with the findings of this study, a cross-sectional study demonstrated higher levels of empathy in younger (17–19 years) as compared to older (20–24 years) medical students. This study discussed the negative correlation between the age of the participants and life satisfaction. This may explain higher empathy levels among young undergraduate medical students^12^.

Medical specialties are broadly categorized into people-oriented and technology-oriented specialties. The former is predominantly focused on people and includes obstetrics/gynecology, internal medicine, pediatrics, and other specialties. A cross-sectional study conducted in Brazil in 2016, demonstrated that students selecting people-oriented specialties have significantly higher empathy mean scores^13^. In our study, people-oriented specialties are associated with relatively higher empathy scores as compared to other specialties.

According to previous studies in Pakistan, it can be inferred that female undergraduate medical students, particularly the students in earlier years of study^11^, which is contradicting our study, demonstrate an initial decline in empathy levels, followed by an increase in the fourth-year of study. The findings of our study are similar to another study conducted in the region, indicating that the highest level of empathy is demonstrated by fourth-year medical students^14^. The empathy trends observed in Pakistan-based studies are different compared to empathy levels among undergraduate medical students in other regions to a certain extent. For instance, a similar study conducted in Saudi Arabia indicated that second-year medical students had the highest empathy levels compared to other years of study^15^. The results of our study not only add to the available pool of evidence regarding undergraduate empathy levels but also emphasize the importance of the increased provision of education and awareness among medical students so as to make them empathetic future healthcare practitioners.

Through the acquisition of empathy scores with regards to these two key variables, this survey is henceforth, able to make relative comparisons and reflect the changing patterns of levels of empathy amongst undergraduate students along their yearly transition from medical students to residents and eventually consultants. This study also takes into account the role of sex in assessing the levels of empathy as well as other relevant factors. The data highlights that the empathy levels of undergraduate Pakistani medical students are significantly lower compared to those enrolled abroad. This finding is consistent and prominent across previous studies in the medical literature, giving rise to the question regarding the differences in the enrollment criteria between national and internal medical schools.

Our study had several limitations. Firstly, its cross-sectional design and the possibility of social desirability bias may have impacted the responses collected via the questionnaire. This may be addressed in future studies where a smaller sample of undergraduates are focused upon and are followed up for a given period so as to preserve the validity of reported outcomes. Secondly, the measurement of empathy scores was self-reported. These scores measure medical students’ empathy but might not be in correlation with their behavior. In addition, religious beliefs and preferences were not considered in our study. Religion dispenses with subjects that are closely related to medicine, such as the suffering of the human body and the cement of life and death, in addition to being strongly connected with spirituality^13^.

In the future, it should be investigated whether nurturing empathy skills is associated with or has an impact on student career preferences and if an association is found between the two, potential strategy implementations regarding medical curriculum and resource allotment could be possibly driven by regional or societal requirements for medical students as well as primary care clinicians.

## Conclusions

This study is the first to demonstrate empathy levels in medical students with respect to sex as well as specialties. Such findings reflect the differences in perceptions of emotions among both sex and were largely consistent with prior literature. We observed relatively less empathy amongst Pakistani medical students in contrast to those enrolled abroad, reflecting the way the learning and working environment impacts these levels leading to burnout and eventually a decline in emotional intelligence. Additionally, we noticed a definite change in empathy levels through the transition from preclinical to clinical years. Furthermore, students pursuing emergency medicine, neurology, obstetrics/gynecology, and oncology as their specialty of choice demonstrated the highest empathy levels followed by pediatrics and internal medicine. The disparity in emotional competencies amongst medical students during their initial years largely needs to be addressed and further evaluated, with regards to the reforms being taken to inculcate activities in their present curriculum in order to enhance their emotional quotient so as to enable them to become better, more responsible clinicians.

## Ethical approval and consent

Ethical approval was obtained from the Institutional Review Board of Ziauddin University, Karachi, Pakistan. Consent was taken from the study participants prior to the acquisition of data. (Protocol reference number 0110721MNY5).

## Sources of funding

None.

## Author contribution

M.N.: conceptualization and project administration; M.N. and Z.N.: data curation; R.F.: methodology/formal analysis/validation; S.Q., B.T.: writing; A.S., W.A., S.A., M.A.: review and editing.

## Conflicts of interest disclosure

There are no relevant financial or nonfinancial competing interests to report.

## Research registration unique identifying number (UIN)

https://clinicaltrials.gov/ct2/show/NCT05736276?term=EMPATHY&cntry=PK&draw=2&rank=1

## Guarantor

Masooma Naseem.

## Data availability statement

Available upon reasonable request.

## Provenance and peer review

Not commissioned, externally peer reviewed.

## Acknowledgements

None.
